# A Rare Case of Chronic Lymphocytic Leukemia and Chronic Myeloid Leukemia in an Elderly Patient: A Case Report

**DOI:** 10.1002/ccr3.73136

**Published:** 2026-10-12

**Authors:** Mostafa Kamandi, Mohammad Mahdi Alvandi Fard

**Affiliations:** ^1^ Hematologist‐Oncologist, Department of Internal Medicine, Faculty of Medicine Mashhad University of Medical Sciences Mashhad Iran; ^2^ Student Research Committee, Faculty of Medicine Mashhad University of Medical Sciences Mashhad Iran

**Keywords:** chronic lymphocytic leukemia, chronic myeloid leukemia, oncology, synchronous malignancy

## Abstract

Chronic Lymphocytic Leukemia (CLL) and Chronic Myeloid Leukemia (CML) are two distinct hematological malignancies with different pathophysiologies and clinical courses. While each is relatively common in the elderly population, their simultaneous occurrence in a single patient is exceptionally rare and poses diagnostic and therapeutic challenges. We present the case of a 79‐year‐old male who was evaluated for progressive fatigue, weight loss, and marked leukocytosis. Laboratory investigations, peripheral blood smear, flow cytometry, and molecular studies were utilized to establish the diagnosis. Initial findings revealed extreme leukocytosis, anemia, and elevated LDH. Peripheral smear and molecular testing confirmed CML through detection of the *BCR::ABL1* fusion gene. Concurrently, flow cytometry identified a clonal B‐cell population positive for CD5, CD19, and CD20, consistent with CLL. Imaging showed splenomegaly and pathologic lymphadenopathy. The patient was treated with imatinib and hydroxyurea, resulting in normalization of white blood cell count, though anemia and thrombocytopenia persisted. This case highlights a rare synchronous diagnosis of CML and CLL in an elderly patient. The comprehensive diagnostic evaluation was critical in establishing the dual pathology. The case underscores the need for heightened clinical suspicion in atypical presentations and contributes to the limited body of knowledge on coexistent myeloid and lymphoid malignancies.

## Key Clinical Message

Simultaneous occurrence of CML and CLL is exceedingly rare; careful integration of molecular and immunophenotypic studies is essential for accurate diagnosis and appropriate management in patients with atypical hematologic presentations.

## 1 Introduction

Chronic Myeloid Leukemia (CML) is a clonal disorder of hematopoietic stem cells, characterized by the presence of a specific chromosomal translocation, *t* (9;22) (q34;q11), which generates the so‐called Philadelphia chromosome (Ph), denoted as 22q–. This cytogenetic abnormality results in the fusion of the ABL1 gene on chromosome 9 with the BCR gene on chromosome 22, producing the *BCR::ABL1* fusion gene. The resulting fusion protein exhibits constitutive tyrosine kinase activity, driving uncontrolled cell proliferation. While the molecular basis and downstream signaling pathways of CML are well elucidated, the initial events that trigger this translocation remain unclear [1]. Chronic Lymphocytic Leukemia (CLL) is defined by the clonal expansion and progressive accumulation of mature B lymphocytes, most of which express the CD5 antigen. These cells infiltrate the peripheral blood, bone marrow, lymphatic tissues, and spleen. The initiating leukemogenic event is thought to occur at the level of multipotent hematopoietic stem cells with self‐renewal capacity, although the precise pathogenesis remains an area of active investigation [2]. Chronic Lymphocytic Leukemia (CLL) is the most prevalent form of leukemia in adults across Western countries. Its incidence is comparable between Europe and the United States, estimated at approximately 4–6 cases per 100,000 individuals annually. In 2014, around 15,720 new cases were projected in the U.S., alongside over 12,000 new diagnoses within Europe. The likelihood of developing CLL increases substantially with age, with over 70% of patients diagnosed at 65 years or older [3].

However, coexistence of CML and CLL in a single patient is exceedingly rare, and there are few documented cases of this in the medical literature, with only a limited number of cases reported in the medical literature. Documented presentations include both sequential diagnoses, where one malignancy follows the other, and synchronous onset, in which both entities are identified at the same time [4]. In most of the documented cases, CML has been diagnosed either following or preceding a diagnosis of CLL. Simultaneous presentation of both malignancies is exceptionally uncommon. The underlying pathogenesis of this rare co‐occurrence remains poorly understood. It is still under debate whether CML and CLL in such cases originate from a common hematopoietic progenitor cell or represent independent clonal proliferations arising through separate oncogenic events [5].

In this Study, we present the case of a 79‐year‐old male who was concurrently diagnosed with CLL and CML, highlighting the clinical presentation, diagnostic workup, and therapeutic approach. This case underscores the importance of comprehensive evaluation in patients presenting with atypical hematological findings and contributes to the limited body of knowledge on the co‐existence of CLL and CML.

## 2 Case Presentation

A 79‐year‐old male with no significant past medical history presented with a 6‐month history of mild fatigue and generalized weakness that initially remained stable but progressively worsened over the past month. He described episodes of his legs “giving out,” along with new‐onset muscle cramping. Additional symptoms included dizziness, unintentional weight loss, night sweats, early satiety, anorexia, and nocturia. He denied any recent fever. Two weeks before admission, he was evaluated by his primary care physician, who noted anemia and marked leukocytosis, prompting referral to our facility for further investigation.

On initial laboratory evaluation at the time of admission, the patient exhibited marked leukocytosis (WBC: 292.74 × 10^3^/μl, with 42% Neutrophils, 24% Lymphocytes, 2% Monocytes, 1% Eosinophils, 1% Basophils, 8% Band Cells, 5% Metamyelocytes, 12% Myelocytes, 1% Promyelocytes and 4% Blast), accompanied by normocytic anemia (Hb: 8.5 g/dL; MCV: 86.3 fl) and elevated RDW (18.2%). The platelet count was within normal limits (343 × 10^3^/μl). The leukocytosis was striking and highly suggestive of a myeloproliferative disorder. Additionally, elevated lactate dehydrogenase (LDH: 890 U/L) suggested increased cell turnover, which is commonly seen in proliferative hematologic malignancies such as CML. Renal and liver function tests were within acceptable ranges, with mild elevations in urea (34 mg/dL) and ALP (203 U/L). Coagulation parameters were normal. Viral hepatitis markers (HBsAg, anti‐HCV, anti‐HBc) were negative (Table 1).

**TABLE 1:** Laboratory tests on‐admission and remission.

On admission		Final evaluation	Reference range	Unit
*Hematology*		*Hematology*		
White Blood Cell (WBC)	292.74	10.7	4–10	10^3^/μL
Neutrophils	42		47–76	%
Lymphocytes	24	82.2%	16–43	%
Monocytes	2		6–9	%
Eosinophiles	1		3–7	%
Basophiles	1		2–3	%
Band Cells	8			%
Metamyelocytes	5			%
Myelocytes	12			%
Promyelocytes	1			%
Blast	4			%
Red Blood Cell (RBC)	3.15	2.81	4.5–5.9	10^6^/μL
Hemoglobin (HB)	8.5	7.5	12.3–15.3	g/dL
Hematocrit (HCT)	27.2	23.8	36–45	%
Mean Corpuscular Volume (MCV)	86.3	84.7	80–96	fL
Mean Corpuscular Hemoglobin (MCH)	27.0	26.7	26.5–32.5	pg
Mean Corpuscular Hemoglobin Concentration (MCHC)	31.3	31.5	30.9–35.5	g/dL
RDW‐CV	18.2	16.3	11.5–15	%
Platelets (PLT)	341	69	150–450	10^3^/μL
*Coagulation profile*				
Prothrombin Time (PT)	15.4		11–13	Sec
International Normalized Ratio (INR)	1.22		0.8–1.2	
Partial Thromboplastin Time (PTT)	41.5		25–35	Sec
*Biochemical Panel*		*Biochemical Panel*		
Urea	34	44	17–45	mg/dL
Creatinine	1.2	1.0	0.7–1.4	mg/dL
Calcium	8.1		8.5–10.5	mg/dL
Phosphorus	5.0		2.5–4.5	mg/dL
Magnesium	2.1		1.8–2.6	mg/dL
Albumin	3.6		3.5–5.5	g/dL
AST	24		5–40	U/L
ALT	19		5–40	U/L
ALP	203		< 258	U/L
Lactate Dehydrogenase (LDH)	890	363	< 530	U/L
Total Bilirubin	1.2		0.3–1.3	mg/dL
Direct Bilirubin	0.4		< 0.3	mg/dL
CRP	1		0–10	mg/L
Sodium	142	139	135–145	mEq/L
Potassium	4.4	4.4	3.5–5.3	mEq/L

### 2.1 Diagnostic Approach

A peripheral blood smear (PBS) revealed a markedly increased number of white blood cells with a full spectrum of myeloid precursors, including myelocytes, metamyelocytes, promyelocytes, and occasional blasts (Figure 1). These findings were highly suggestive of chronic myeloid leukemia (CML). Also, lymphocytes accounted for 24% of the total white blood cells. Given the marked leukocytosis (WBC: 292.74 × 10^3^/μl), this corresponds to an absolute lymphocyte count of 70.26 × 10^3^/μl, which is significantly elevated. Although the relative percentage of lymphocytes falls within the normal range, the markedly increased absolute count further supports the presence of a proliferative hematologic disorder. Due to the markedly elevated myeloid cells in peripheral blood smear, the patient was started on Hydroxyurea from the first day. Over an 18‐day course of treatment, his leukocyte count declined significantly to 11.8 × 10^3^/μl.

**FIGURE 1:**
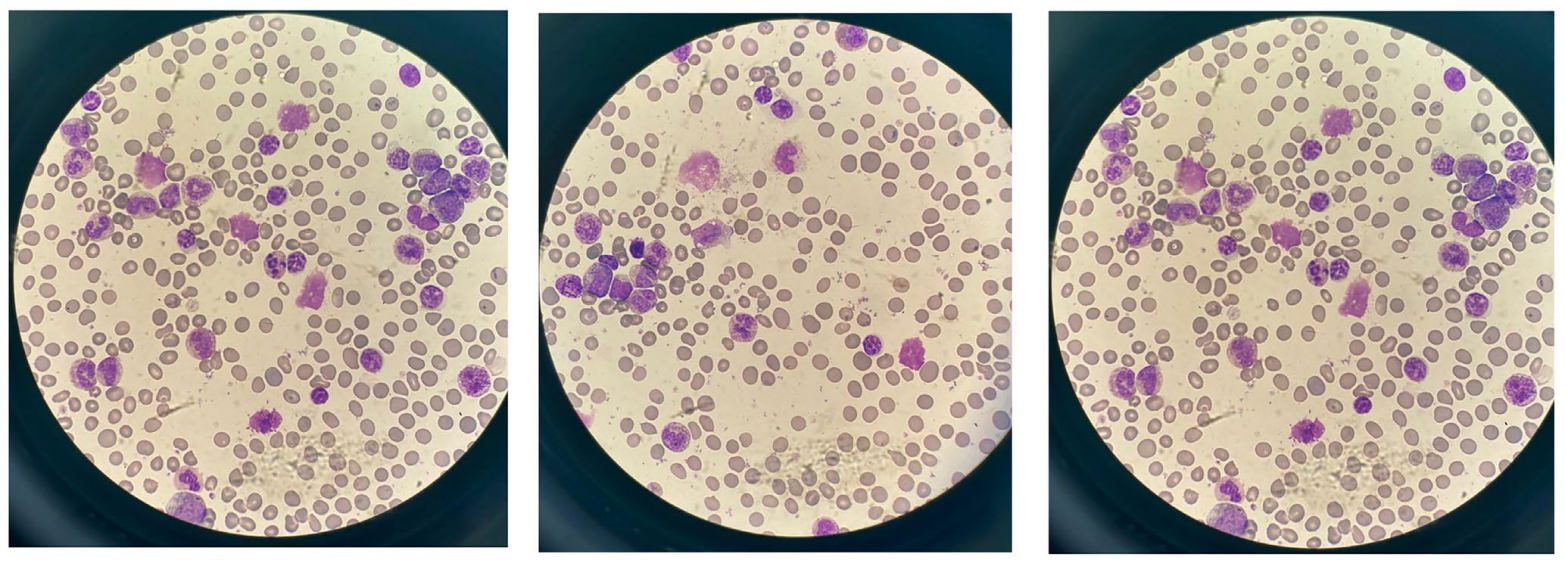
Peripheral blood smear (PBS) consistent with Chronic Myeloid Leukemia.

To confirm the diagnosis of CML, BCR‐ABL molecular testing was requested. Additionally, flow cytometry of peripheral blood was performed as part of the diagnostic workup and to assess the blast percentage. Molecular studies revealed strong expression of the *BCR::ABL1* p210 transcript and weak expression of p190, confirming the diagnosis of CML. Given that peripheral blood smear findings were predominantly suggestive of CML, the patient was started on imatinib (from day 10 of admission). In contrast, Further evaluation with flow cytometry of peripheral blood demonstrated a clonal B‐cell population expressing CD5, CD19, and CD20, consistent with chronic lymphocytic leukemia (CLL). The test was repeated for confirmation and yielded identical results.

### 2.2 Imaging Studies

Abdominal and pelvic ultrasonography identified multiple pathologic abdominal lymphadenopathies. Portacaval nodes measured up to 15 mm in short‐axis diameter, and left mesenteric nodes up to 12 mm. The lymph nodes were round, lacked a central echogenic hilum, and exhibited a pathological appearance. Splenomegaly was also noted, with a cranio‐caudal diameter of 205 mm.

At the time of discharge, leukocyte count had further normalized (WBC: 10.7 × 10^3^/μl); however, anemia had worsened (Hb: 7.5 g/dL), and thrombocytopenia had developed (platelets: 69 × 10^3^/μl), possibly due to disease burden or cytotoxic therapy. Lymphocyte percentage was markedly elevated (82.2%), supporting the diagnosis of a concurrent lymphoproliferative disorder. LDH had decreased (363 U/L), indicating reduced cellular turnover (Table 1).

### 2.3 Final Diagnosis

The diagnosis of concurrent chronic myeloid leukemia and chronic lymphocytic leukemia was established based on characteristic peripheral smear findings, immunophenotyping by flow cytometry, and molecular confirmation of *BCR::ABL1*. This rare coexistence of a myeloproliferative and lymphoproliferative neoplasm poses significant diagnostic and therapeutic challenges. The patient was discharged in a clinically stable condition with a prescription for imatinib and a scheduled hematology follow‐up.

The patient was started on imatinib at a dose of 400 mg daily. After 2 months of continuous treatment, a significant clinical and hematological response was observed. A follow‐up complete blood count revealed a marked reduction in the previously elevated white blood cell count. However, persistent lymphocytosis remained. The patient's general condition and performance status improved notably during this period. Unfortunately, approximately 2 months after the initiation of treatment, the patient developed severe community‐acquired pneumonia and subsequently died due to respiratory failure. No further follow‐up was possible beyond this point.

## 3 Discussion

This case documents the rare concurrent presentation of chronic myeloid leukemia (CML) and chronic lymphocytic leukemia (CLL) in a 79‐year‐old male. The patient presented with constitutional symptoms, marked leukocytosis, and anemia, with subsequent diagnostic studies confirming the simultaneous presence of both myeloid and lymphoid neoplasms. This unusual coexistence of distinct hematological malignancies presents significant diagnostic and therapeutic challenges that require careful consideration in clinical management.

### 3.1 Epidemiology and Clinical Significance

The simultaneous occurrence of CML and CLL in a single patient represents an exceedingly rare phenomenon in hematologic oncology. CLL is the most commonly diagnosed leukemia in adults in Western countries, particularly affecting older individuals, while CML is the most common myeloproliferative disorder characterized by the *BCR::ABL1* fusion gene [6]. Despite their prevalence in elderly populations, their coexistence is extremely uncommon, with only approximately 62 documented cases in the literature [7]. The pattern of presentation in our patient aligns with what has been described as “simultaneous occurrence” of both conditions. According to the literature, the coexistence of CML and CLL can be classified into three main patterns: CML preceding CLL, CLL preceding CML, and simultaneous occurrence, with the latter being the least common form [6, 7]. Among previously reported cases, seven patients were documented to have developed CML and CLL simultaneously [7].

### 3.2 Pathophysiological Mechanisms

Several hypotheses have been proposed for this biclonal leukemic presentation, though the precise pathophysiology remains unclear.

#### 3.2.1 Independent (“Biclonal”) Origin

Cytogenetic and molecular studies in some cases show that the two malignancies arise from separate hematopoietic clones. For example, Maher et al. demonstrated *BCR::ABL1* fusion only in the myeloid/granulocytic fraction, whereas the monoclonal immunoglobulin gene rearrangement was confined to the lymphoid (CLL) cells [8] Such findings support two distinct malignant clones coexisting rather than a single clone transforming along two lineages.

#### 3.2.2 Common Stem‐Cell Origin

Conversely, some authors speculate that both neoplasms may originate from a single pluripotent hematopoietic stem cell that acquires mutations enabling both myeloid and lymphoid transformation [9]. This is supported by the idea that *BCR::ABL1* could occur in an early progenitor, theoretically giving rise to both myeloid (CML) and lymphoid (CLL) clones. A common genetic “hit” could explain concurrent disease in the same individual, although direct proof is lacking.

#### 3.2.3 Microenvironmental and Cytokine Effects

Another proposed mechanism is that leukemic cells of one lineage create a milieu that promotes the other malignancy. Studies have shown that *BCR::ABL1* positive cells produce cytokines such as interleukin‐3, which can stimulate CD34^+^ progenitors and B‐lymphoid cells [9]. In theory, a CML clone could secrete factors that drive expansion of a B‐lymphoid clone (or vice versa). However, this remains speculative.

#### 3.2.4 Therapy‐Related or Immune Dysregulation

Chronic lymphocytic leukemia is known to predispose patients to second malignancies, often thought to arise from immune dysfunction or prior treatment [4]. In over half of the reported cases, CLL preceded CML, and many of those patients had received chemoimmunotherapy (e.g., fludarabine, chlorambucil). It has been suggested that DNA damage or selection pressures from CLL therapy might contribute to a subsequent CML. Conversely, prolonged immune dysregulation in CLL could facilitate a second hematologic neoplasm. However, in simultaneous cases (with no prior treatment), this is less clear. Likewise, our patient had no history of cytotoxic therapy.

Given the conflicting data, the consensus is that the pathogenesis is heterogeneous. In some reported patients, the two leukemias demonstrably arise from separate clones [9, 10], whereas others are best explained by a shared stem‐cell origin. It may be that several factors—a predisposing genetic instability, environmental triggers, or stochastic events—contribute to this rare dual leukemogenesis. Importantly, no single unifying mechanism has been confirmed, and further research is needed to understand how and why these two diseases occur.

## 4 Conclusion

This case highlights the rare and challenging coexistence of Chronic Myeloid Leukemia (CML) and Chronic Lymphocytic Leukemia (CLL) in a single patient, which has only been reported in a few instances in the literature. The patient presented with symptoms suggestive of both myeloproliferative and lymphoproliferative disorders, leading to a comprehensive diagnostic workup. The peripheral blood smear, flow cytometry findings, and molecular studies were pivotal in confirming the diagnosis of both CML and CLL, a phenomenon that raises questions regarding the pathogenesis and potential common progenitor cell origin of these two hematologic malignancies. While the underlying mechanism remains unclear, this case underscores the importance of thorough diagnostic evaluation in atypical presentations. Management of such coexisting conditions requires careful consideration, and ongoing research is needed to better understand the molecular basis and optimal management strategies for patients with these rare concurrent hematologic disorders.

## Author Contributions

**Mostafa Kamandi:** conceptualization, project administration, writing – original draft. **Mohammad Mahdi Alvandi Fard:** writing – original draft, writing – review and editing.

## Funding

The authors have nothing to report.

## Consent

Written informed consent was obtained from the patient for publication of this case report and any accompanying images.

## Data Availability

Data sharing not applicable to this article as no datasets were generated or analyzed during the current study.
